# Supplementary material for: Prebiotic and Anti-Adipogenic Effects of Radish Green Polysaccharide
Source: Microorganisms. 2023 Jul 24;11(7):1862. doi: 10.3390/microorganisms11071862 (PMC10385334; doi:10.3390/microorganisms11071862)
Supplement: Supplementary file 1 [file microorganisms-11-01862-s001.zip › microorganisms-2499993-supplementary.pdf]

Table S1. Specific contents of three short chain fatty acids (SCFAs).

Probiotic Strain	Group	Acetic Acid (μM)	Propionic Acid (μM)	Butyric Acid (μM)	Total SCFAs (μM)
L. paracasei	NC	47.80 ± 6.84	5.32 ± 1.36	5.55 ± 1.75	58.67 ± 9.95
	Inulin	9.77 ± 1.60	0.52 ± 0.06	0.62 ± 0.23	10.91 ± 1.31
	PRG 0.1%	122.65 ± 128.14	5.69 ± 4.71	3.11 ± 3.44	131.44 ± 136.29
	PRG 0.5%	132.84 ± 130.40	5.45 ± 2.77	1.14 ± 0.75	139.43 ± 133.92
L. plantarum	NC	130.61 ± 79.54 ^{c}	6.12 ± 1.05 ^{c}	5.34 ± 2.48 ^{a}	142.07 ± 76.01 ^{c}
	Inulin	56.67 ± 8.69 ^{d}	6.46 ± 4.41 ^{c}	0.78 ± 0.35 ^{b}	63.91 ± 13.45 ^{c}
	PRG 0.1%	424.87 ± 127.5 ^{b}	15.94 ± 0.21 ^{b}	1.72 ± 0.38 ^{b}	442.53 ± 128.13 ^{b}
	PRG 0.5%	1011.87 ± 49.72 ^{a}	26.19 ± 5.62 ^{a}	2.23 ± 0.36 ^{b}	1040.29 ± 55.70 ^{a}
L. lactis	NC	139.49 ± 19.16 ^{c}	5.41 ± 0.23 ^{c}	2.62 ± 0.64 ^{b}	147.53 ± 18.28 ^{c}
	Inulin	75.42 ± 23.21 ^{d}	3.66 ± 0.69 ^{c}	2.34 ± 2.46 ^{b}	81.43 ± 20.06 ^{d}
	PRG 0.1%	329.81 ± 83.89 ^{b}	13.12 ± 0.33 ^{b}	7.04 ± 7.81 ^{b}	349.96 ± 91.38 ^{b}
	PRG 0.5%	768.90 ± 19.91 ^{a}	16.52 ± 0.78 ^{a}	29.54 ± 1.71 ^{a}	815.01 ± 22.39 ^{a}
B. bifidum	NC	54.33 ± 19.45 ^{c}	3.22 ± 1.73 ^{b}	2.27 ± 0.10 ^{c}	59.82 ± 21.08 ^{c}
	Inulin	256.81 ± 38.99 ^{b}	4.62 ± 0.22 ^{b}	7.77 ± 2.18 ^{b}	278.20 ± 40.95 ^{b}
	PRG 0.1%	269.01 ± 98.18 ^{b}	4.66 ± 3.84 ^{b}	3.76 ± 0.87 ^{c}	277.43 ± 102.90 ^{b}
	PRG 0.5%	558.33 ± 6.53 ^{a}	11.07 ± 0.12 ^{a}	39.41 ± 25.42 ^{a}	608.80 ± 19.01 ^{a}
B. longum	NC	73.41 ± 9.27 ^{b}	5.98 ± 0.79	2.29 ± 1.38	81.67 ± 7.11 ^{b}
	Inulin	231.93 ± 34.36 ^{a}	6.42 ± 0.40	12.82 ±13.63	251.17 ± 48.39 ^{a}
	PRG 0.1%	264.52 ± 131.35 ^{a}	5.05 ± 0.66	9.36 ± 0.56	278.93 ± 131.24 ^{a}
	PRG 0.5%	423.68 ± 273.55 ^{a}	6.78 ± 0.78	2.45 ± 0.07	432.91 ± 274.25 ^{a}

The different letters indicate significant difference ($p < 0.05$) determined by Duncan's multiple range test.
